# The Open Physiology workflow: modeling processes over physiology circuitboards of interoperable tissue units

**DOI:** 10.3389/fphys.2015.00024

**Published:** 2015-02-24

**Authors:** Bernard de Bono, Soroush Safaei, Pierre Grenon, David P. Nickerson, Samuel Alexander, Michiel Helvensteijn, Joost N. Kok, Natallia Kokash, Alan Wu, Tommy Yu, Peter Hunter, Richard A. Baldock

**Affiliations:** ^1^Centre for Health Informatics and Multiprofessional Education, University College LondonLondon, UK; ^2^Auckland Bioengineering Institute, University of AucklandAuckland, New Zealand; ^3^Leiden Institute of Advanced Computer Science, University of LeidenLeiden, Netherlands; ^4^Medical Research Council Human Genetics Unit, Institute of Genetics and Molecular Medicine (IGMM), University of EdinburghEdinburgh, UK

**Keywords:** functional tissue units, histology, physiology-based pharmacokinetics, fluid flow modeling, physiology circuit-boarding, ApiNATOMY, visual knowledge management

## Abstract

A key challenge for the physiology modeling community is to enable the searching, objective comparison and, ultimately, re-use of models and associated data that are interoperable in terms of their physiological meaning. In this work, we outline the development of a workflow to modularize the simulation of tissue-level processes in physiology. In particular, we show how, via this approach, we can systematically extract, parcellate and annotate tissue histology data to represent component units of tissue function. These functional units are semantically interoperable, in terms of their physiological meaning. In particular, they are interoperable with respect to [i] each other and with respect to [ii] a circuitboard representation of long-range advective routes of fluid flow over which to model long-range molecular exchange between these units. We exemplify this approach through the combination of models for physiology-based pharmacokinetics and pharmacodynamics to quantitatively depict biological mechanisms across multiple scales. Links to the data, models and software components that constitute this workflow are found at http://open-physiology.org/.

## Introduction

The cataloging of molecular function, for the purpose of organizing model and data resources in molecular biology, has given rise to powerful global maps describing [i] the type of functions that molecules contribute to processes in the body, as well as [ii] how these functions relate to one another. Two such catalogs of key importance take the form of controlled vocabularies in ontological form: namely [i] the Gene Ontology's Molecular Function (Gene Ontology Consortium et al., 2013) ontology (**GO_MF**), describing the function of gene products, and [ii] the Chemical Entities of Biological Interest (**ChEBI**) ontology (Hastings et al., 2013), describing the function of small molecules. Crucially, all terms from these two ontologies imply that an intermolecular binding interaction process has to occur for the function of a molecule to be realized.

A key goal for the physiology community is to build upon the above ontological maps (i.e., GO_MF and ChEBI), ensuring that tissue-level representations of function are coherent with molecular ones. In effect, ensuring this coherence is to explicitly relate tissue function to molecular function.

In this paper, we describe the development of a tissue knowledge management (**KM**) workflow that combines histology image analysis with multiscale modeling of flow processes to explicitly represent and articulate this relationship. We refer to this approach as the Open Physiology workflow^1^. In particular, we shall focus on applying this KM workflow to model the influence that tissues play on molecular function by regulating when and where intermolecular binding occurs. In practice, the quantitative study of this regulation is achieved by modeling the role of tissue in orchestrating the co-location of interactors within the same diffusive compartment.

Primarily, our driving use case is the modularised modeling e.g., as discussed in Petersen et al. (2014) of drug absorption, distribution, metabolism and elimination (**ADME**) by multiple tissues (e.g., intestine, kidney, liver). In effect, these tissues heavily influence when and where a drug co-locates with its transporters, metabolizing enzymes and target receptors in the same diffusive compartment. However, given that our goal is to provide a general solution for the bridging of molecular and tissue function in physiology and pathophysiology, we also take into account molecular synthesis (rarely a consideration for drugs) as a key addition to the ADME spectrum, and refer to the resulting set of processes as **ADMES**.

In practice, achieving the above goal of modeling molecular transitions within and between diffusive compartments entails overcoming two formidable representational challenges of tissue structure and function, namely, how to:

1. apportion tissue space in terms of diffusive parcellations (i.e., a parcel of tissue within which any two points are within diffusion distance of one another) to enable the modeling of transitions in molecular co-location, driven by Brownian motion, across subcellular compartments inside and across nearby cells, as well as
2. connect these parcellations across distances that are well beyond the diffusion limit (e.g., to describe the transfer of oxygen molecules from lung tissue to skeletal muscle tissue in the thighs) to enable modeling of fluid flow that conveys molecules from one organ to another, or across distant tissue regions within the same organ.

This paper describes, for the first time, the Open Physiology workflow that overcomes the above two challenges to generate data and models that coherently bridge diffusive and advective processes in support of ADMES modeling. We make use of the term “circuitboard” to refer to the knowledge we have generated to bridge these processes, as well as the circuitboard-style graphics we use to create a visual depiction of this knowledge.

The Methods section describes the core technical components of the workflow. In particular, we show how we generate tissue parcellations from 3D histology images, known as primary Functional Tissue Units (**pFTUs**) (de Bono et al., 2013; Hunter and de Bono, 2014), and link these units to models of long-range fluid flow to study drug ADMES. The Results section discusses the data on pFTUs and anatomical routing, and the KM tools that make use of this data to study ADMES processes coherently. In the Discussion, we focus on [i] the implication of this Open Physiology effort on ADMES studies in general and drug ADME in particular, as well as [ii] the technical priorities that inform the roadmap for our future work.

## Materials and methods

Our work was carried out over three distinct steps (outlined respectively in the section titled “Representing and Articulating Multiscale Tissue Knowledge to Modeling Fluid Flow and its Effect on Drug Distribution” below). The first two steps were carried out in parallel, i.e., the curation of circuitboard knowledge for short- and long-range flows (Section Representing and Articulating Multiscale Tissue Knowledge), and the generation and annotation of tissue unit image data (section titled “Generating Tissue Parcellation Data”). The output of these two steps provided the starting point for the modeling effort, discussed in the section titled “Modeling Fluid Flow and its Effect on Drug Distribution”.

### Representing and articulating multiscale tissue knowledge

#### Managing ontology-based knowledge of anatomy over multiple scales

The primary technical goal for this module of the workflow is to build a multiscale topological representation of anatomical compartments over which to model advective and diffusive flow. A key requirement for this topological representation is that the semantic standard of its knowledge is interoperable with that adopted by the community generating the catalogs of molecular function (i.e., GO_MF and ChEBI) discussed in the Introduction. To this end:

1. for the semantic annotation of the components of this topological model, we have selected ontologies maintained by the Open Biomedical Ontologies (**OBO**) community (Smith et al., 2007) and adopted by the Virtual Physiological Human (Hunter et al., 2013) community as standard for resource annotation. In particular, these ontologies include: the Foundational Model of Anatomy (**FMA**) (Rosse and Mejino, 2003) to describe gross anatomy, the CellType ontology (**CT**) (Bard et al., 2005) for cellular entities, and the Gene Ontology's Cellular Component (**GO_CC**) ontology for subcellular structures;
2. for the management of the resulting topological data, and corresponding semantic annotation metadata, we have applied two software modules, namely: (i) RICORDO (de Bono et al., 2011; Wimalaratne et al., 2012) for annotation management and automated inferencing over the above ontologies to classify and query resulting semantic metadata, as well as (ii) ApiNATOMY (de Bono et al., 2012) for the visual browsing and interaction with graphical depictions of ontologies, semantic metadata that make use of these ontologies, and topology data.

In particular, the RICORDO knowledgebase component was populated with the ontologies discussed in #1 above, and the ApiNATOMY tool was set to articulate and visualize these ontologies together with the corresponding semantic metadata holdings that are managed by the RICORDO module.

#### Developing and managing knowledge about routes of flow

The scope for the explicit knowledge representation of conduits conveying molecules beyond the diffusive limit covers three main types of vessel:

1. Multicellular Endothelial: e.g., blood vessels, lymphatic vessels;
2. Multicellular Epithelial: e.g., airways, biliary tree, urinary tract, reproductive tract;
3. Unicellular: e.g., neuron.

**Figure 1:**
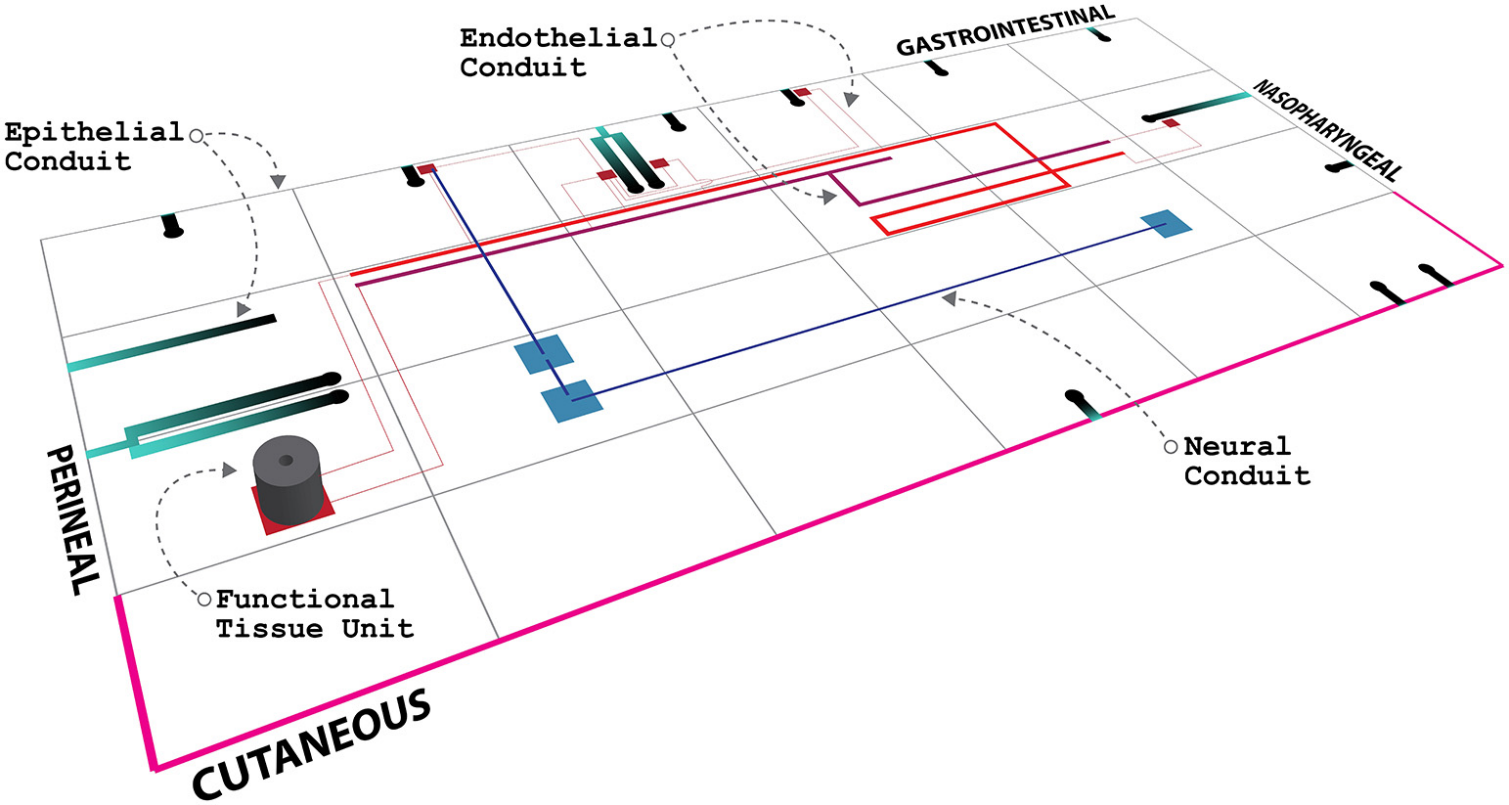
**Mock-up of an ApiNATOMY circuitboard display, showing the anatomical layout of a tiled depiction of body regions, together with an edge-based illustration of advective conduits, as well as a cylindrical pFTU**.

An illustration of an ApiNATOMY view of routes conveyed by the above types of vessels (together with an *in situ* depiction of a pFTU) is shown in Figure 1. In this work, we focus on the development of a topological model for blood vessel connections, in view of these vessels' anatomical ubiquity and relevance to most types of ADMES study.

The cardiovascular knowledgebase in version 3.1 of the FMA was extracted. This knowledge consisted of terms representing named vessels and their connections. The number of arteries listed in this resource was 3833, and the number of veins was 2151. In addition, a total of 266 organ regions in the FMA were found to be linked to be “supplied” or “drained” by at least one artery or one vein respectively.

**Figure 2:**
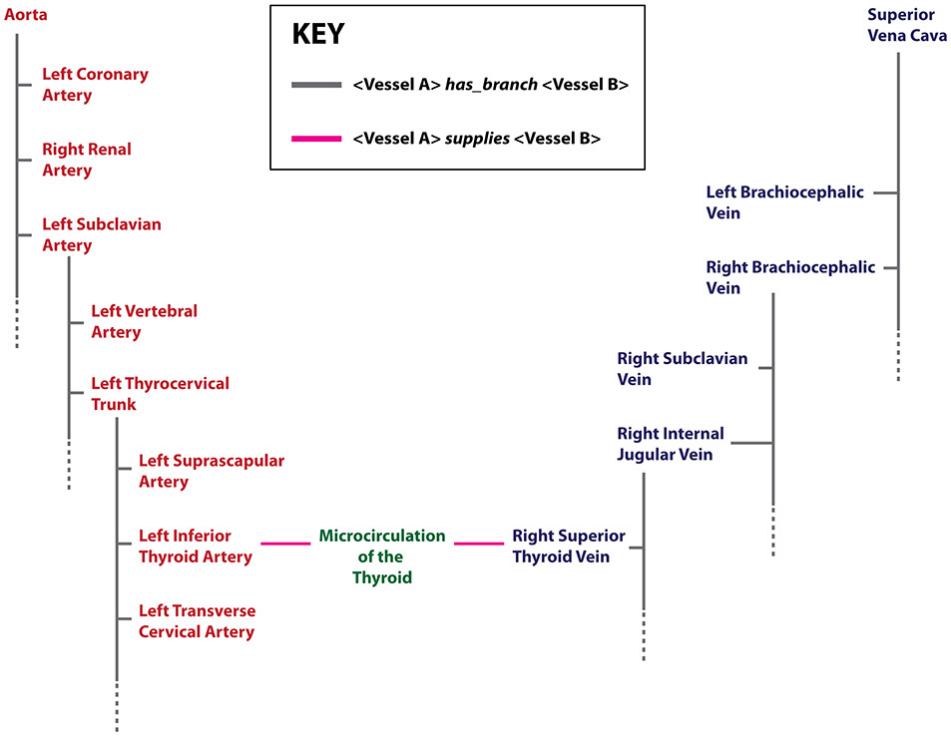
**An example of terms and relations relevant to cardiovascular knowledge representation in the VKB**. Arterial terms are shown in red, venous terms in blue. In this diagram, an organ region that is supplied or drained by arteries or veins respectively is labeled as “Microcirculation” and shown in green. Two key relations (depicted as edges) are shown: (i) the *has_branch* (gray) that associates a parent vessel with its branches—the dotted gray line indicates that only a subset of the branches of a parent vessel is shown, and (ii) the *supplies* (pink) relation indicates the provision and drainage of blood between organ microcirculations and connected large vessels.

New vessel terms and new connections between vessels were curated to create a vascular knowledgebase (**VKB**). In particular, two key vascular knowledge representation additions to the FMA were added, namely: [i] a more granular representation of small blood vessels involved in the microcirculation of organs and their tissues, and [ii] the explicit representation of the order by which branches originate from their parent vessel (Figure 2).

The above two extensions were required to ensure the detailed representation of routes of connectivity in the cardiovascular system. The solution adopted relied on the expression of vascular knowledge in a logic-based language, the Knowledge Interchange Format (**KIF**)^2^, that supports spatial knowledge representation and reasoning. This step, therefore, formalized a core ontology of the vascular system that is designed to support the representation of topological aspects of large blood vessels and the microcirculations they supply or drain. This logic-based knowledge representation of vascular anatomy was implemented in KIF using version 4.0 of the PowerLoom application ^3^. The KIF representation was primed by importing the VKB, and could be queried using both classification and rule-based inferencing.

In the KIF representation, the whole of the known cardiovascular system is represented as a connected series of vessel segments, each segment having an explicit semantic annotation to an FMA term. A vessel segment represents either:

1. an unbranched region of a large blood vessel (e.g., the region of the Left Common Carotid Artery between its origin from the aorta to the point where Left External Carotid Artery branches off), or
2. one of three components of microcirculation. Mircocirculation components are defined as a serial connection between [a] an aggregate of arterioles, [b] an aggregate of capillaries and [c] an aggregate of venules, in this order.

#### Applying histology templates to acquire pFTU knowledge

The geometric definition of a pFTU is described in de Bono et al. (2013): a pFTU consists of a cylindrical diffusive field with an 80-micron diameter and 60-micron length. The long axis of this field is aligned on a central advective vessel—the types of vessel involved in a pFTU are listed in the section titled “Developing and managing knowledge about routes of flow”. In process modeling scenarios involving fluid flow, these central vessels are connected to topological representations of routes of long-range advection (e.g., capillary components in microcirculation, as described above). From a modeling perspective, therefore, the above structural configuration of a pFTU represents the point of transition from advection- to diffusion-driven processes.

**Figure 3:**
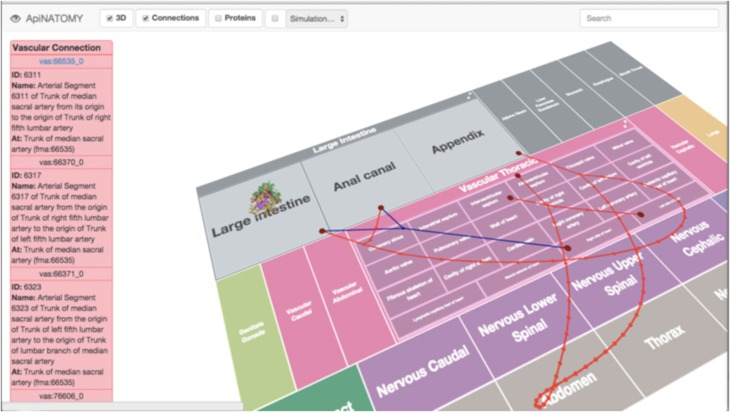
**A screenshot from the ApiNATOMY circuitboard user interface, showing an example of cellular spatial distribution in a pFTU derived from the human colon**. Overlay of red edges represents arterial connections, and venous connections are in blue.

Templates conforming to the above geometric configuration are applied to the acquisition of 3D tissue image data, as discussed in the section titled “pFTU annotation”. The minimum annotation requirement for pFTU image data includes [i] a term from the FMA to indicate the organ region from which the tissue sample was derived, and [ii] terms from CT to indicate a non-redundant list of distinct cell types that are within the same diffusive field. We refer to the combination of an FMA term and the corresponding list of CT terms as the primary tissue motif (**PTM**) for a particular pFTU. The ontology-based annotation arising from pFTU analysis can be stored in the RICORDO and visualized in ApiNATOMY (Figure 3).

### Generating tissue parcellation data

#### Sample preparation and imaging

We obtained human tissue samples in glutaraldehyde fixative, and rinsed them in a cacodylate buffer and cut into approximately 3 mm pieces suitable for plastic embedding. Each piece was then dehydrated in increased concentrations of ethanol (10, 30, 50, 70, 85, 95) at 10-min intervals then finally 3 times at 100%. After dehydration each piece was transferred into propylene oxide (100%) for 2 × 10 mins then into 1:3, 1:1, 3:1 resin/propylene oxide mixtures for 10 min each before being placed in a thin layer of complete resin overnight at room temp in the fume hood to start infiltrating.

Each was given a second overnight infiltrating step in fresh resin before embedding in more fresh resin in an aluminum mold and allowed to polymerize for 48 h at 60°C in oven in fume hood. Blocks were then cut out and trimmed for sectioning on the LKB V ultratome with diamond knife.

The first block was cut to produce a stack of 270 serial sections at 2 microns over 8 slides. Slides were then stained in 0.5% Toluidine Blue made up in 0.5% borax at 60°C for 25 s in a water bath, washed in distilled water and allowed to air dry. Finally slides were mounted in Zeiss immersion oil using No 1.5 thickness coverslips.

Each histological section was imaged using a x20 objective on the Olympus DotSlide slide scanner. This step delivers 2D images generated from a series of individual captures (patches) that are shade-corrected and “stitched” together to a single large image. In total, 270 sections were cut and imaged, corresponding to a 540-micron thick segment of the gut lining and the resulting 270 images passed on for 3D reconstruction.

#### 3D image reconstruction

The plastic embedded histological sections do not suffer significant distortion from the processing stages and therefore the section images are realigned by pair-wise matching to establish the rigid-body affine transform between each adjacent image. The process is fully automatic. Each adjacent pair of images is aligned to minimize the sum of squared gray-value differences over the entire image using the ITK^4^ image library. The first pass alignment will occasionally give rise to a poor match between images so is checked by comparing the squared gray-value difference against a profile of expected differences arising from nearby matches. If a large variance is detected then the alignment is repeated using a broader search space with random seed start points until the difference from the gray-difference profile is reduced. If that does not work the images are reviewed manually and individual sections may need to be removed from the stack. This is typically due to gross artifacts such as obscuring dirt, bubbles or torn sections. This secondary alignment uses the Woolz (Husz et al., 2009) image-processing library with bespoke software for managing the parameter space search.

#### pFTU annotation

**Figure 4:**
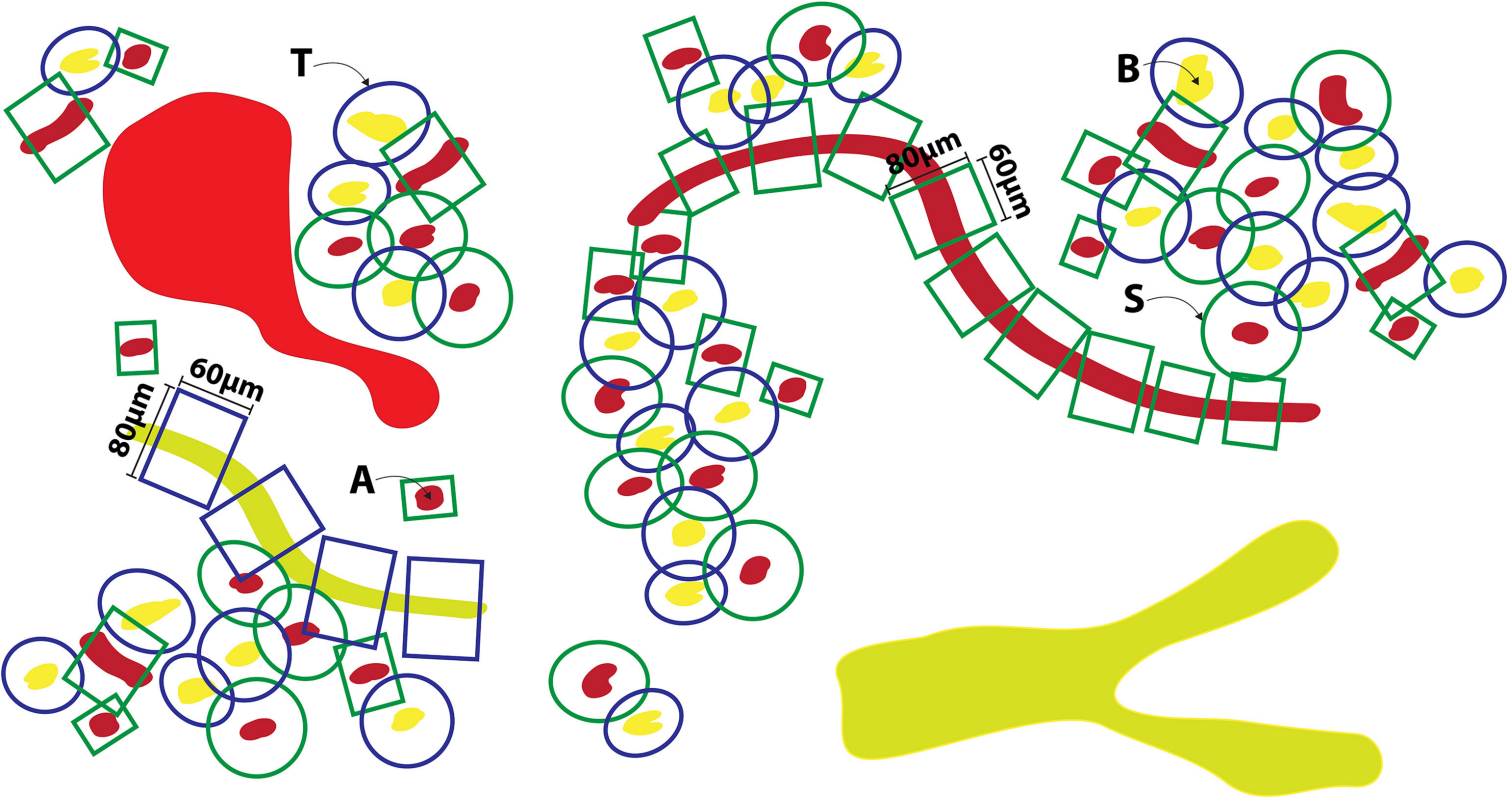
**Illustrating the application of templates to acquire 3D spatial pFTU data from a block of epithelial tissue (e.g., pancreas)—the schematic view of a 2D section through this block is illustrated here**. [A] Red, capillary vessels; [B] Yellow, epithelial conduits (e.g., pancreatic acinar ducts); [S], Endothelial pFTU; [T] Epithelial pFTU.

**Figure 5:**
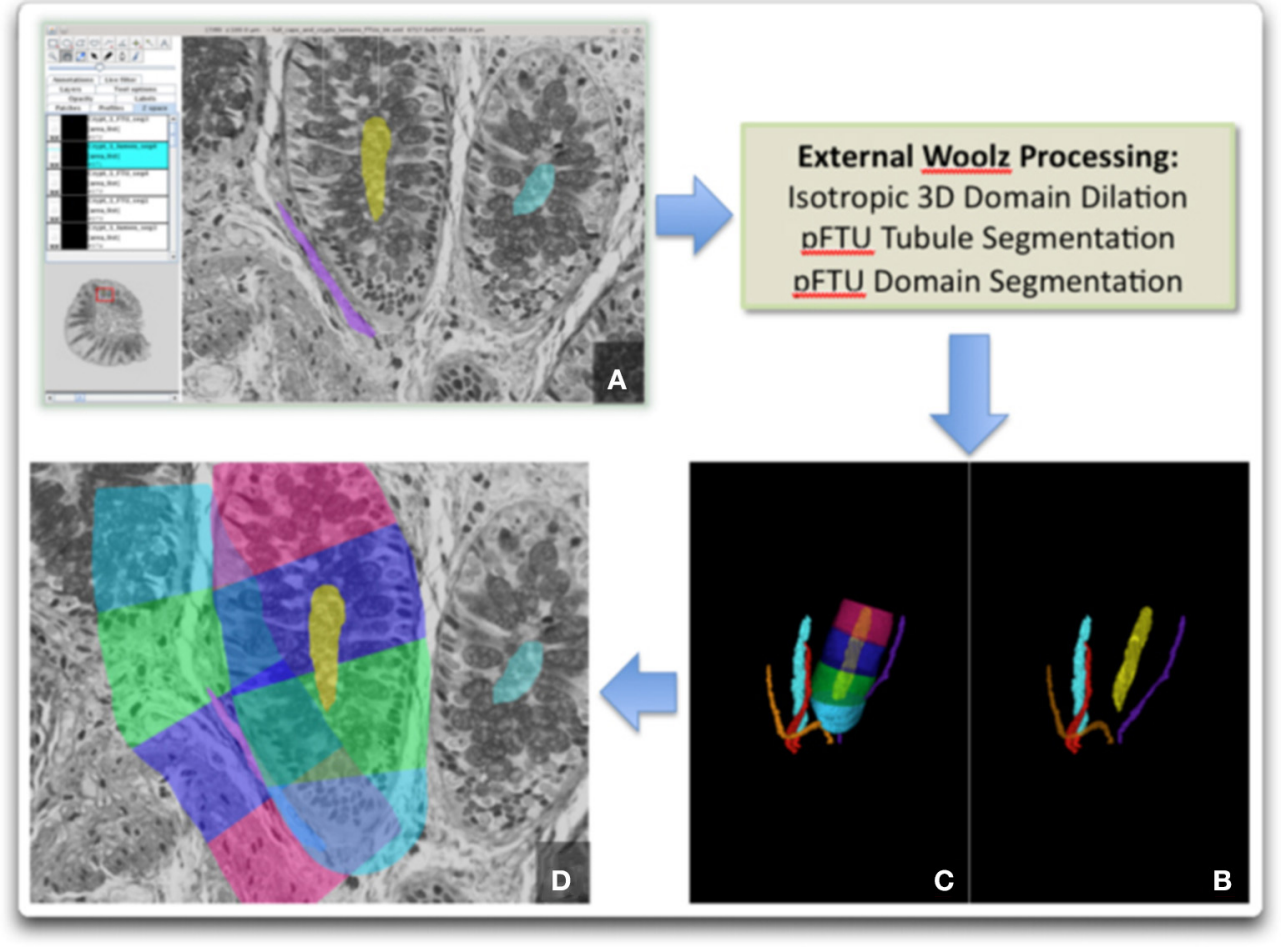
**Image processing sequence to generate the pFTU domains**. External processing with Woolz is required for 3D domain dilation and pFTU modeling: **(A)** Fiji-TrakEM2 window showing primary capillary and colonic crypt delineation; **(B)** 3D domains for the capillaries and crypt lumens; **(C)** pFTU regions for one of the colonic crypts; **(D)** 2D section view showing the pFTU regions superimposed on the histology and illustrating spatial overlap of pFTUs of different type (i.e., in this case, endothelial and epithelial).

A schematic illustration of the application of the pFTU templates, discussed in the section titled “Applying histology templates to acquire pFTU knowledge”, to histology image data is depicted in Figure 4. The central advective channels were first delineated manually using the ImageJ TrakEM2 software (Cardona et al., 2012). The reconstructed image stack is formatted as a series of 2D jpeg format images and read in as a “virtual stack” to TrakEM. The software is designed to allow manual delineation of structures, which are connected, as contiguous domains through the 3D space. Within ImageJ these can be visualized as 3D structures (Figure 5). To define the FTU domain within a 40-micron radius of the channel, the structures are exported as labeled volume images and then segmented to individual channel spatial domains using the Woolz image processing software (WlzThreshold). These domains are then dilated using a structuring element defined as a 40-micron sphere, appropriately scaled to take account of the non-isotropic voxel sampling in 3D in the reconstruction. This operation using WlzStructDilate is fast and could not be achieved within ImageJ except as an approximation using incremental dilation, which would not give the true Euclidean distance dilation. The pFTU domain for the full central advective conduit (e.g., a capillary) was then cut into 60-micron segments using the MAPaint application. This allows planar cut of domains at any orientation and for this purpose the plane was set perpendicular to the tubule at each end of the 60-micron length. Figure 2 also shows the result of this action. The cut regions are then read back into ImageJ as labeled images for visualization and further processing.

#### Cell type annotation and visualization

**Figure 6:**
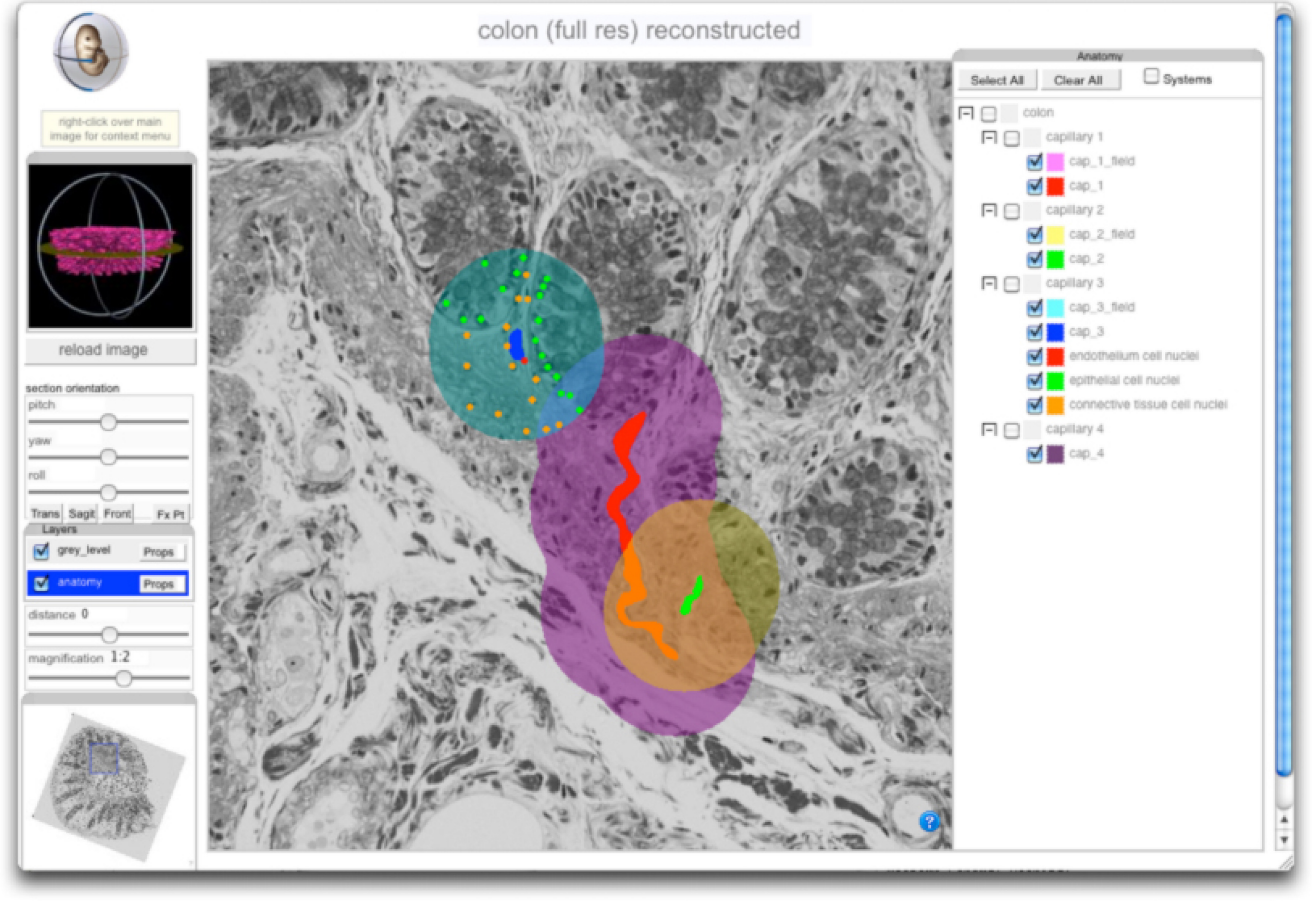
**Screenshot of GUI for the 3D reconstruction of pFTU- and PTM-annotated human colonic mucosa**. Viewer controls allow arbitrary re-sectioning through the reconstructed volume, shown here with overlaid domains associated with 3 capillaries (left to right: painted blue, red and green) and their associated pFTUs (left to right: painted turqoise, purple and orange). The leftmost domain also shows CT cell annotation. The viewer shows how, in general, cells can be members of multiple pFTUs. The viewer is available at Cardona et al. (2012).

With the pFTU domains defined, and each annotated with an FMA term, an expert histopathologist then marks the individual cells manually by way of annotation with CT terms. For this we used the MAPaint application, which allows rapid marking of cell nuclei of any given type (Figure 6). The results of these annotations can then be used to analyse the tissues in terms of the PTMs and for their spatial distributions of cells in normal and diseased tissues (de Bono et al., 2013)^5^ (e.g., as shown in Figure 3).

To make the 3D volumetric images with FTU domain and cell mark-up visible, we have used the IIP3D technology (Husz et al., 2012) to deliver section-views of the underlying image data with an overlay of the image annotation. The IIP3D server delivers a tile-based viewer and the overlay domains can be selected dynamically to allow visual analysis of the overlapping regions and the cells that are typically shared between multiple pFTUs due to overlap between diffusive fields (as depicted in Figure 1). The viewer has been developed using Ajax/Javascript and will run on any modern web-browser.

### Modeling fluid flow and its effect on drug distribution

The flow modeling strategy is to link (i) FMA-annotated segments from the topological model of blood vessel connections in the VKB to (ii) the central advective channel of FMA- and CT-annotated pFTUs generated from the parcellation analysis described above. This linkage is assembled through the RICORDO annotation, and visualized via the ApiNATOMY graphical user interface (**GUI**).

In practice, the flow modeling approach makes use of one-dimensional time-varying Navier-Stokes equations that incorporate the elastic properties of the vascular wall via a nonlinear pressure-area relation (Safaei, 2014). The vascular model includes all segments in the VKB, and lumped parameter CellML (Cooling et al., 2008) models of the vascular bed impedance are used to connect the arterial and venous sides over microcirculation components that include pFTU modules. The ontology terms that annotate microcirculation components are derived from the PTM associated with pFTU data. In particular, this step highlights a critical role of PTM annotation, because PTM information ensures:

1. the correct correspondence of (arterial and venous) vasculature model to microcirculatory model for a specific body region, through a correspondence of FMA terms, and
2. the legitimacy of combining cell-specific models (i.e., models that are annotated with CT terms) to study tissue-level processes, given that such cells are provably within diffusion distance in the same pFTU.

**Figure 7:**
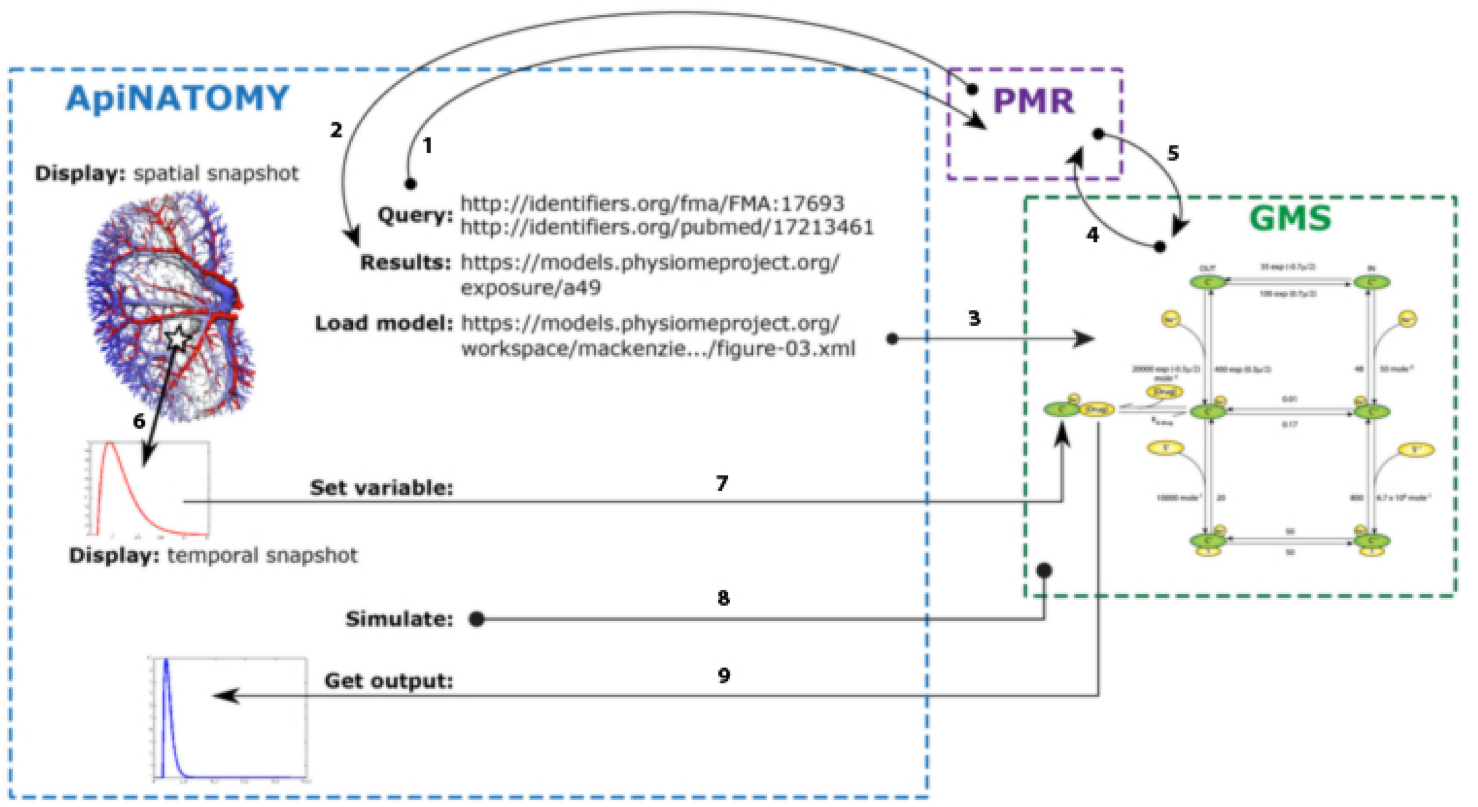
**Illustrative example of the interaction between ApiNATOMY, PMR web services, and the GMS to generate the GUI output shown in Figure 8**. Using the SPARQL^9^ endpoint provided by the RICORDO services implemented in PMR, ApiNATOMY is able to execute SPARQL queries using the PMR metadata repository [arrow 1, in bold]. In the example shown, ApiNATOMY is querying for a given FMA term (for the renal proximal tubule) and a specific paper identified via a PubMed ID. PMR responds to the query providing all matching PMR exposures [arrow 2], from which the ApiNATOMY user selects the appropriate PMR workspace (identified by the URL shown in the diagram). From the selected workspace, the ApiNATOMY user selects a specific CellML model (or the tool infers the required CellML model from information obtained from the exposure definition in PMR) and the GMS is instructed to load that CellML model [arrow 3]. Upon receiving this instruction, the GMS will request the model from PMR (Petersen et al., 2014) and instantiate that model into an internal executable form (de Bono et al., 2013). ApiNATOMY is able to sample spatial fields (Hunter and de Bono, 2014) to extract temporal snapshots for a specific spatial location. Using services provided by the GMS, ApiNATOMY is able to select a particular variables in the instantiated CellML model (Smith et al., 2007; Rosse and Mejino, 2003) and instruct the GMS to use the temporal snapshot to define that variable (Smith et al., 2007). This service requires the transfer of the temporal snapshot from ApiNATOMY to the GMS using a standard JavaScript array encoded as a JSON string^10^, ^11^. Once a particular simulation is fully defined, ApiNATOMY instructs the GMS to execute the simulation (Hunter et al., 2013), over the time interval specified by the central timing module. Following the execution of the simulation, ApiNATOMY requests the simulated variable transient(s) for the desired model variables (Rosse and Mejino, 2003) and presents the results to the user. Once again, this data is transferred as JavaScript arrays encoded in the JSON format.

The above combination of advection- and diffusion-driven models is supported by the PMR platform (Yu et al., 2011), an extensible software system that provides the infrastructure for collaborative development and sharing of models, that supports basic reasoning over the annotations of its component models. The PMR can be trawled to extract reference to all models, their components and any annotatable object, in particular, model variables. This step results in the ascription of stable IDs (linked to permanent URLs that are used as Uniform Resource identifiers in RICORDO) to these objects and the constitution of a corresponding metadata repository (Figure 7).

Once the pressure and flows have been computed throughout the cardiac cycle, an advection-diffusion equation is solved in order to compute the concentration of a drug or other ligand (e.g., hormone, cytokine) throughout the vascular system and throughout the cardiac cycle. For instance, the introduction of a drug at any point in the vascular system can be defined as a time-dependent and distributed boundary condition (e.g., uptake from the digestive tract) or as a time-varying point source (e.g., directly via a needle). The result of this computation is then a time-varying concentration of the drug in every tissue of the body. This computed transient tissue drug concentration provides an input to combinations of cell models encoded in CellML that share annotations with PTMs. The Physiome Model Repository^6^ contains many examples of tissue processes that can be coupled to the vascular model via ligand binding receptors.

**Figure 8:**
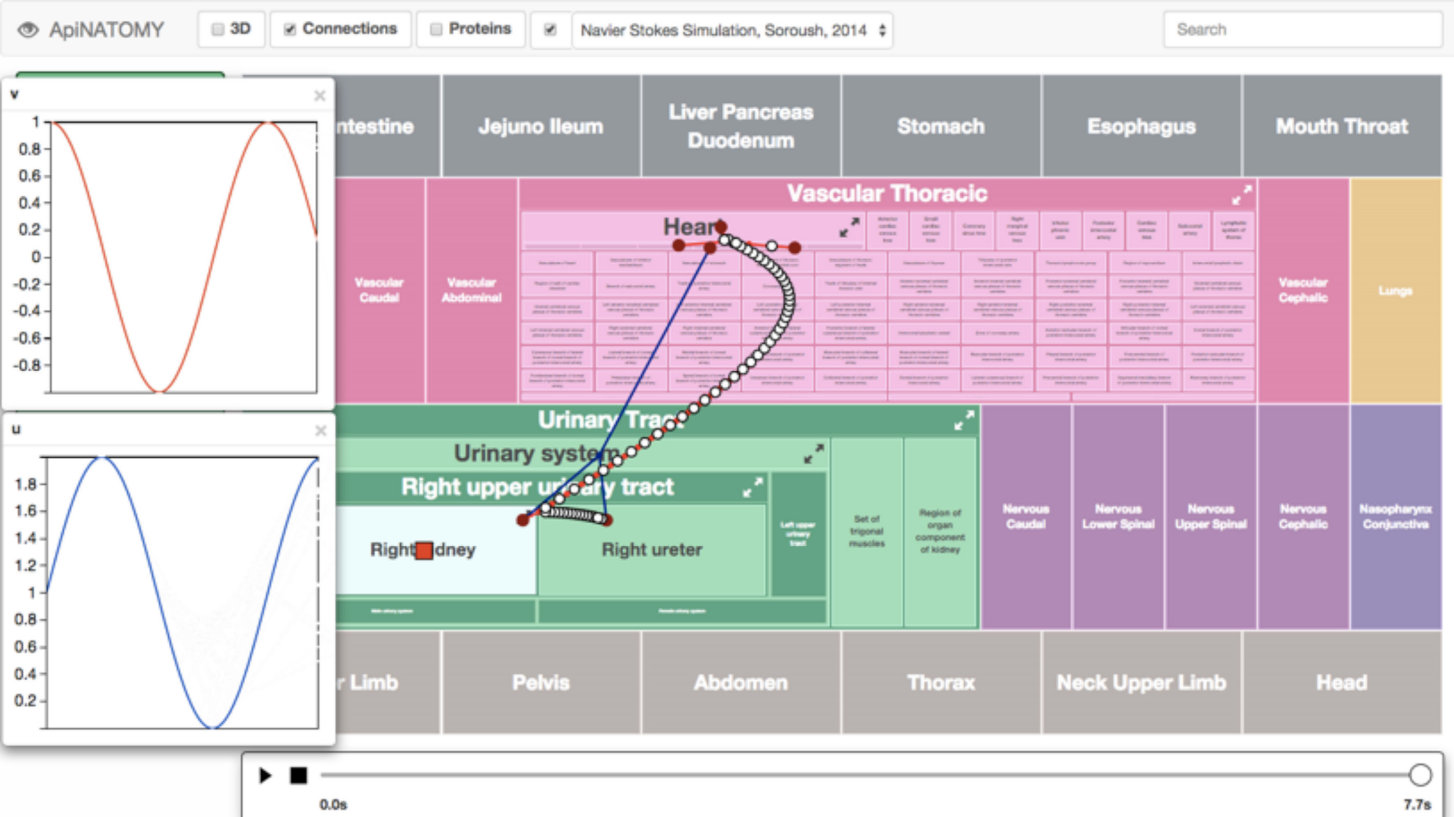
**Screenshot of an ApiNATOMY GUI displaying time-varying cyclical changes in blood pressure [v] and flow [u] in the kidney microcirculation**. The arterial (red) and venous (blue) vascular routes connecting the heart to the kidney is also overlaid onto the circuitboard. A red box glyph representing RICORDO semantic metadata annotation is located in the Right Kidney tile (i.e., the location of the glyph represents the annotation of the model variable to the Right Kidney term in the FMA), and a number of glyphs are overlaid on the blood-vessel representations connecting the Right Upper Urinary Tract to the Heart. These glyphs are rendered on an SVG layer (scalable vector graphics), and each represents a specific variable. Clicking on them brings up a graph plotting that variable over time. The time dimension can be manipulated and traversed with the slider at the bottom.

Flow is modeled through the vascular network using OpenCMISS (Bradley et al., 2011). Time varying solutions for pressure, flow, drug concentration and drug effect are computed from the relevant models and exported to ApiNATOMY for display on the semantic graphs corresponding to the relevant anatomical region (Figure 8).

Simulations for the effect on tissue of the hypothetical drug are carried out on a GET model server (**GMS**—documentation and code available at^7^, ^8^) a standalone server that provides web-services for interacting with CellML models. The services relevant here are those related to the model simulation service. The steps linking the execution of the simulation of CellML models using GMS to the ApiNATOMY GUI are illustrated in Figure 7.

The ApiNATOMY component contains a central timing module to control and synchronize dynamic model content, such as certain simulations and animations. The timer can run in real-time, or be manually controlled through a slider-bar. By interfacing with the GET model server, ApiNATOMY allows direct interaction with CellML models, synchronized through the timing module. The semantic metadata repository associated with the PMR allows ApiNATOMY to discover the various variables of a CellML model, and display their traces in a line-chart, or set of line-charts. These charts can also show ‘alternate timeline’ traces for comparison purposes, and will show exact values on mouse-over of glyphs representing these variables in the correct anatomical context. Mouse-over on graph depictions of blood vessels, also gives rise to displays of location-specific pressure, flow and drug concentration data.

## Results

**Figure 9:**
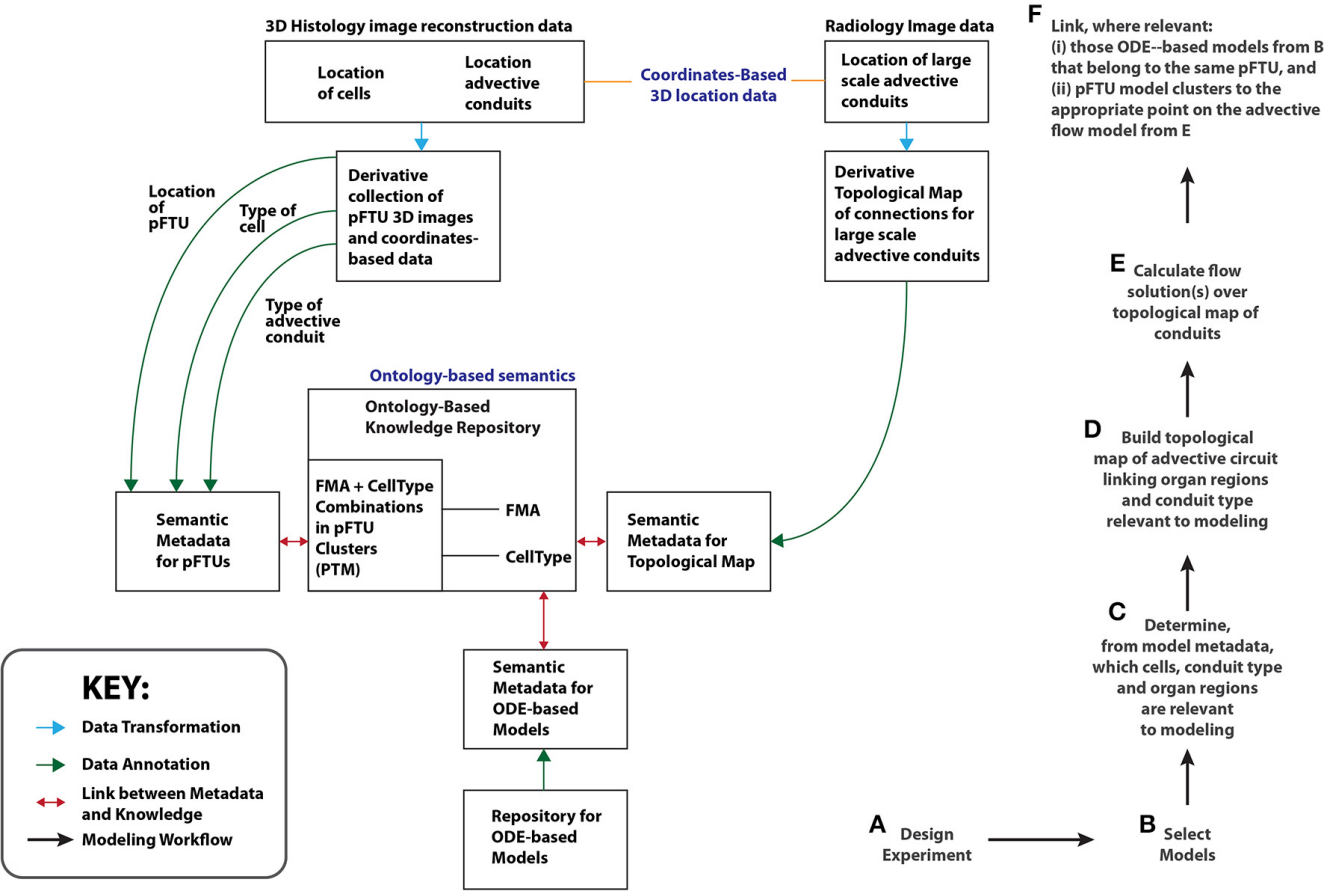
**A schematic illustrating the Open Physiology modeling workflow (Right: steps A–F)**. On the left of the diagram, the relationships between the key resources relevant to this workflow are also shown. An example of a topological map is the Vascular KnowledgeBase (VKB) referred to in the section titled “Developing and Managing Knowledge About Routes of Flow”. The term PTM stands for Primary Tissue Motif, discussed in the section titled “Applying Histology Templates to Acquire pFTU Knowledge”. The RICORDO tool manages the mapping between semantic metadata and ontology-based knowledge (i.e., the double-headed red arrows). ApiNATOMY automatically generates (i) treemaps out of ontology-based knowledge, and overlays (ii) edges from topological maps to create a circuitboard. Glyphs representing semantic metadata in RICORDO are then graphically overlaid onto the circuitboard.

The main result reported in this paper is the implementation of a workflow (summarized in Figure 9) that brings together components that deal with the:

1. application of ontology-based KM methods to standardize the knowledge representation of [a] routes of fluid flow across the body, as well as [b] the anatomical location and cellular content of tissue parcellations. In particular, the KM framework we describe ensures the semantic interoperability and combination of route knowledge with pFTU knowledge. Critically, the basis for this semantic interoperability is drawn from ontology-based annotation standards already adopted by the computational physiology community;
2. experimental generation and ontology-based semantic annotation of pFTU parcellation data derived from 3D histology image reconstruction;
3. combination of the results from #1 and #2 above to model advective and diffusive flows that affect molecular interaction.

To implement a histology component of this workflow, we have developed a prototype pipeline that captures the pFTU structural detail for physiological modeling. To capture the required detail, we have used thin serial sectioning and 3D reconstruction from individual section images. This allows the use of standard histological staining which provides a more complete representation of the tissue. Other techniques can supplement this approach, in particular immunohistochemistry (**IHC**) for specific proteins. However, the IHC approach *per se* may not be able to deliver the tissue detail required for all modeling requirements. In this paper, we showed how the pipeline may be used to define pFTU domains associated with endothelial and epithelial conduits. The individual advective channels are defined manually, followed by a semi-automatic process to cut the channel to 60-micron lengths and define the associated diffusive domains.

The ApiNATOMY circuitboard GUI permits the visual interaction with the flow route topological data, as well as the blood flow-related calculations' parameter settings and output, providing a useful modeling dashboard in an multiscale anatomical context.

All data, models and results generated from the above work will be available via the Open Physiology website at http://open-physiology.org/.

## Discussion

Computational physiology has achieved significant progress in developing mathematical approaches that describe physiology processes in terms of molecular transitions across multiple scales. In so doing, this field has generated an impressive array of models that simulate these events. However, this work has also given rise to considerable fragmentation and redundancy. This shortcoming is partly due to either poverty or incoherence in the explicit representation of multiscale anatomical location within these models, or their associated data. In practice, the explicit representation of anatomical location associated with physiology models is either too poorly described to begin with or, when anatomy is described, it is depicted by non-standard means (e.g., as free text comments, rather than by ontology-based annotation). This limitation has led to an inadequate search capability of community-shared modeling resources (i.e., giving rise to duplication of effort, as relevant resources cannot be found), as well as a lack of interoperability (i.e., leading to fragmentation, as models cannot talk to one another) when resources *are* found.

Given the above obstacle, the Open Physiology approach is to provide the technical means to explicitly assemble circuits of multiscale anatomy in terms of ontologies of biological structure and, in so doing, coherently link models of molecular processes that are annotated by terms from these ontologies. Furthermore, the generation of ApiNATOMY circuitboards that depict the route of molecular flow across the body provides the computational means to represent molecular transitions across anatomical compartments across multiple scales.

### Addressing the challenge of bridging molecular and tissue functions

In practice, the open physiology workflow has three key implications to admes studies:

1. **physiology models and data may be automatically connected over a route map**
  A key goal for the Open Physiology effort is the annotation with terms from reference ontologies, linked to the circuitboard, with which to imbue model elements (e.g., variables, parameters) with precise meaning in terms of anatomical location. This coherent application of annotation provides the means to automatically infer the physiological relationship between ADMES models (or their corresponding data) over [i] location parthood, [i] subclass, as well as [iii] topology of flow route. In practice, our aim is to provide robust tools for annotation and automated inferencing [discussed in de Bono et al. (2011) and Wimalaratne et al. (2012)] that further support and incentivise the annotation of data and model resources [e.g., CellML Cooling et al. (2008), SBML Chelliah et al. (2013)] by the community. Critically our goal is to foster the unfettered and open sharing of this type of annotation to make resources easy to find by the community.
2. **the representation of molecular transitions across tissues is made explicit**
  The pFTU approach to tissue parcellation provides a rational scaffold for the ADMES modeling of molecular exchange processes across compartments. In particular, this approach lends itself to combining models to study the direct effect of tissue structure on molecular function, including models about the regulation of:
  - phospholipid membrane permeability (e.g., plasma membrane);
  - permeability of the lining (e.g., endothelial gaps, basement membrane) of the central advective vessel with respect to the parenchymal cuff around it;
  - flow rate along the central advective vessel, as a function of one-dimensional time-varying calculations over topological representations of long-range advective conduits;
  - the degree of overlap between the diffusive fields from neighboring pFTUs (as illustrated in Figure 5D), as a function of tissue structure alteration resulting from processes such as development, growth, as well as physiological or pathological adaptation (e.g., Rodríguez-Gómez et al., 2013; Gonçalves et al., 2014).
3. **efforts in drug discovery may be bridged to drug development**
  The field that focuses on discovering drug leads is heavily reliant on the management of tissue-specific knowledge about molecular pathways (e.g., projects like OpenPHACTS attest to this reliance^12^). The assembly of this type of knowledge results from the combination of [i] tissue-specific expression data with [ii] knowledge of molecular transfer and transformations across different subcellular compartments curated from a wide range of experiments [e.g., Orchard et al. (2014) and Matthews et al. (2009)]. To this end, the Open Physiology workflow will provide a well-defined biophysical scaffold onto which to organize knowledge about tissue-specific molecular pathways, in particular:
  - the application of pFTU templates will facilitate the acquisition of 3D spatial IHC data (outlined in the section titled “Results”, above) within pre-defined diffusive fields that cuff central advective vessels;
  - the annotation of PTM data outlines the cellular content in a pFTU—this data is interoperable with the semantic standards of key community pathway resources (i.e., which describe cellular compartments in terms of ontologies drawn from the OBO Foundry resource) enabling the overlay of these pathway data onto PTM compartments;
  - from inception, the pFTU data object can leverage the semantics of its annotation to link to topological representations of long-range conduits. This property of a pFTU allows tissue-level data to be plugged into body-level models of fluid flow (e.g., blood flow), in support of physiology-based pharmacokinetic (**PBPK**) and pharmacodynamics (**PD**) studies of ADME in drug discovery [a mainstay in projects such as DDMoRe (Harnisch et al., 2013)].

### Priorities for our future work

Development on the infrastructure underlying the Open Physiology workflow is in progress, and our next steps are focused on three priority areas, namely:

#### Circuitboarding: routing calculations

A core objective in study of molecular process coordination across distant sites in the body is to characterize ADMES routes through which regulatory influences are conveyed. In practice, the following questions are consistently asked when studying the coordination of two processes that are sited in anatomically distinct locations (in the following example, these locations are labeled A and B):

- via which compartments does a ligand, synthesized in location A, have to transit to reach its target receptor synthesized within location B?
- what other (target or off-target) receptors to the same ligand are encountered *en route* that might affect communication between A to B?

Our next step, therefore, is to leverage inferencing methods over the route knowledgebase to take into account [i] tissue-specific expression data, [ii] molecular interaction data and [iii] permeability data at compartmental boundaries (as discussed in point #2 of the section titled “Addressing the Challenge of Bridging Molecular and Tissue Functions”) to automate the calculation of routes between anatomical locations, and identify relevant influences exerted along the way.

#### pFTUs: automating extraction and statistical modeling

The tissue analysis pipeline described here is semi-automatic and uses a combination of tools as needed for different parts of the workflow. This part of the workflow will be streamlined and developed as a server-based pipeline to allow remote and collaborative delineation of central advective vessels, coupled with the fully automatic generation of pFTU diffusive domains and individual cell annotation. This advance will be achieved by using the IIP3D image server technology^13^ coupled with Javascript-based interfaces to capture manual input and visualize the automatic analysis. The image-processing and cell-recognition modules will use the ImageJ and Woolz image-processing tools to reconstruct and segment the data with defined image domains. The resultant output will be maintained in a database for user review and downstream analysis. This database will provide essential data for statistical modeling of tissue-specific distributions of [i] IHC patterns within pFTUs, as well as [ii] different pFTU types within the same block of tissue. Such models would be applied to histology image classifier training and as parameters for ADMES modeling.

#### ADMES modeling: tissue fields

Our goal is to generate algorithmically data about vascular bed dimensions from the automated segmentation of 3D organ images, coupled to a transmission line model that includes all blood vessels down to 10 μm. At that point, a lumped parameter empirical 0D pressure-flow description of the capillary beds will be used to link the arterial and venous ends of the tissue models of the larger vessels. This blood flow model will link the pFTU-derived virtual tissue modules, generated from the statistical models discussed in pFTUs: Automating extraction and statistical modeling above, to study the ADMES processes via local diffusion and long-range vascular advection. Diffusion models based on partial differential equations (**PDEs**) will be developed for the virtual tissue modules, using 3D tissue IHC data to derive spatial protein expression. These PDE models will then be approximated using homogenisation model reduction techniques to provide an ODE representation of each tissue type that can be inserted into the distributed vascular model, in support of whole-body PBPK and PD modeling ^14^. The cellular processes that will be handled by PBPK ODE models will include a number of core mechanisms in physiology (e.g., pH control, glucose regulation). Each of these processes will be encoded in the VPH/Physiome CellML modeling standard (e.g., ^15^ ^16^ and their ontology-based annotations also publicly shared.

## Conclusion

The Open Physiology approach is also being developed to provide the means for drug and disease experts to explicitly describe multi-site and multiscale ADMES mechanisms in terms of the location and process knowledge depicted on the circuitboard. In particular, it provides a potential avenue to manage complex knowledge about anatomy in direct support of multiscale modeling. Our goal is to continue to enhance the computational physiology effort through the provision of richness and coherence of anatomy location knowledge to match the mathematical rigor and eloquence that already typifies the repertoire of physiology models developed by this community.

### Conflict of interest statement

The authors declare that the research was conducted in the absence of any commercial or financial relationships that could be construed as a potential conflict of interest.

## Footnotes

^1^Open Physiology [Internet]. [cited 2014 Nov 7]. Available from: http://open-physiology.org/

^2^Knowledge Interchange Format [Internet]. [cited 2014 Oct 30]. Available from: http://logic.stanford.edu/kif/dpans.html

^3^PowerLoom Knowledge Representation and Reasoning System [Internet]. [cited 2014 Oct 30]. Available from: http://www.isi.edu/isd/LOOM/PowerLoom/

^4^ITK—Segmentation and Registration Toolkit [Internet]. [cited 2014 Oct 30]. Available from: http://www.itk.org/

^5^[cited 2014 Nov 7]. Available from: http://aberlour.hgu.mrc.ac.uk/eAtlasViewer_demo/application/TPRDemo/wlz/colonRecon.php

^6^Physiome Repository—Physiome Model Repository [Internet]. [cited 2014 Nov 6]. Available from: https://models.physiomeproject.org/welcome

^7^get—Bitbucket [Internet]. [cited 2014 Oct 30]. Available from: https://bitbucket.org/get

^8^GET Documentation—GET 0.1 documentation [Internet]. [cited 2014 Oct 30]. Available from: http://get-documentation.readthedocs.org/en/latest/

^9^SPARQL 1.1 Overview [Internet]. [cited 2014 Dec 21]. Available from: http://www.w3.org/TR/sparql11-overview/

^10^Final draft of the TC39 “The JSON Data Interchange Format” standard - ECMA-404.pdf [Internet]. [cited 2014 Dec 21]. Available from: http://www.ecma-international.org/publications/files/ECMA-ST/ECMA-404.pdf

^11^ECMAScript Language Specification—ECMA-262 Edition 5.1 [Internet]. [cited 2014 Dec 21]. Available from: http://www.ecma-international.org/ecma-262/5.1/

^12^Open PHACTS Home [Internet]. [cited 2012 Oct 16]. Available from: http://www.openphacts.org/

^13^IIP3D (WlzIIP) [Internet]. [cited 2014 Nov 6]. Available from: http://www.emouseatlas.org/emap/analysis_tools_resources/software/wlziip.html

^14^PKPD model of interleukin-21 effects on thermoregulation in monkeys (Pharmacodynamic)—Physiome Model Repository [Internet]. [cited 2014 Nov 6]. Available from: https://models.physiomeproject.org/exposure/504e7681708fc7b1260db25363658be5/overgaard_pharmacodynamic_2007.cellml/view

^15^PKPD—Physiome Model Repository [Internet]. [cited 2014 Nov 6]. Available from: https://models.physiomeproject.org/PKPD

^16^Endocrine—Physiome Model Repository [Internet]. [cited 2014 Nov 6]. Available from: https://models.physiomeproject.org/endocrine
